# Health insurance and contraceptive use, Indonesian Family Planning Census 2021

**DOI:** 10.2471/BLT.22.289438

**Published:** 2023-06-15

**Authors:** Asri Maharani, Sujarwoto Sujarwoto, Mario Ekoriano

**Affiliations:** a Department of Nursing, Faculty of Health and Education, Manchester Metropolitan University, Brooks Building, 53 Bonsall Street, Hulme, Manchester, M15 6GX, England.; b Department of Public Administration, Universitas Brawijaya, Malang, Indonesia.; c Indonesia National Research and Innovation Agency, Jakarta Pusat, Indonesia.

## Abstract

**Objective:**

To assess the association between health insurance coverage and sociodemographic characteristics, and the use of modern contraception in Indonesia.

**Method:**

We used data from the 2021 Indonesian family planning census which included 38 408 597 couples. Contraception is covered by the national health insurance scheme: members are non-contributory (for poor families who do not make any monetary contribution) or contributory (for better-off families who pay for the insurance). We used regression analyses to examine the correlation between each type of health insurance (non-contributory, contributory, private or none) and contraceptive use and type of contraceptive used.

**Findings:**

The prevalence of the use of modern contraceptives in Indonesia was 57.0% (21 897 319/38 408 597). Compared with not having health insurance, having health insurance was associated with a greater likelihood of contraceptive use, odds ratio (OR): 1.14 (95% confidence intervals, CI: 1.13–1.14) and OR: 1.01 (95% CI: 1.01–1.01) for women with non-contributory and contributory health insurance, respectively. Having private health insurance was associated with lower use of modern contraceptives (OR: 0.94; 95% CI: 0.94–0.94). Intrauterine devices, lactational amenorrhoea and tubal ligation were the most common forms of contraceptive used by women.

**Conclusion:**

The prevalence of modern contraceptive use in Indonesia is lower than the 75% target of the 2030 sustainable development goals. As national health insurance positively correlated with modern contraceptive use, extending its coverage on remote Indonesian islands is recommended to increase the use of such contraceptive methods in those areas.

## Résumé

**Objectif:**

Identifier les liens entre couverture d'assurance-maladie et caractéristiques sociodémographiques d'une part, et recours aux moyens de contraception modernes en Indonésie d'autre part.

**Méthodes:**

Nous avons utilisé les données issues du recensement de la planification familiale indonésienne 2021, qui portait sur 38 408 597 couples. La contraception est prise en charge par le régime national d'assurance-maladie: les membres sont soit non contributifs (familles pauvres ne versant aucune participation financière), soit contributifs (familles plus aisées qui paient pour être assurées). Nous avons effectué des analyses de régression afin d'examiner la corrélation entre chaque type d'assurance-maladie (non contributive, contributive, privée ou inexistante), et l'usage ainsi que le type de contraceptif employé.

**Résultats:**

Dans le pays, 57,0% (21 897 319/38 408 597) des couples étudiés utilisaient des contraceptifs modernes. Comparé aux femmes sans assurance-maladie, les femmes ayant une assurance-maladie étaient davantage susceptibles de recourir à des contraceptifs, l'odds ratio (OR) s'élevant à 1,14 (intervalle de confiance de 95%, IC: 1,13-1,14) pour celles bénéficiant d'une assurance-maladie non contributive et à 1,01 (IC de 95%: 1,01–1,01) pour celles bénéficiant d'une assurance-maladie contributive. Par ailleurs, posséder une assurance-maladie privée allait de pair avec un usage moins fréquent de contraceptifs modernes (OR: 0,94; IC de 95%: 0,94-0,94). Les dispositifs intra-utérins, l'aménorrhée durant l'allaitement et la ligature des trompes figuraient parmi les moyens de contraception les plus répandus chez les femmes.

**Conclusion:**

La fréquence d'utilisation de contraceptifs modernes en Indonésie est inférieure au seuil de 75% fixé par les objectifs de développement durable 2030. Comme l'assurance-maladie nationale exerce un impact positif sur l'usage de contraceptifs modernes, il est recommandé d'étendre sa couverture dans les îles indonésiennes isolées afin d'accroître le recours à de telles méthodes dans ces régions.

## Resumen

**Objetivo:**

Evaluar la asociación entre la cobertura del seguro médico y las características sociodemográficas, y el uso de los métodos anticonceptivos modernos en Indonesia.

**Métodos:**

Se utilizaron datos del censo de la planificación familiar de Indonesia de 2021, que incluía a 38 408 597 parejas. La cobertura de la anticoncepción está a cargo del sistema nacional de seguro de enfermedad: los afiliados son no contributivos (para las familias pobres que no hacen ninguna aportación monetaria) y o contributivos (para las familias más acomodadas que pagan el seguro). Se utilizaron análisis de regresión para examinar la correlación entre cada tipo de seguro sanitario (no contributivo, contributivo, privado o ninguno) y el uso de anticonceptivos y el tipo de anticonceptivo utilizado.

**Resultados:**

La prevalencia del uso de anticonceptivos modernos en Indonesia fue del 57,0% (21 897 319/38 408 597). En comparación con no tener seguro médico, tener seguro médico se asoció con una mayor probabilidad de uso de anticonceptivos, riesgo relativo (*odds ratio*, OR): 1,14 (intervalos de confianza del 95%, IC: 1,13-1,14) y OR: 1,01 (IC del 95%: 1,01-1,01) para mujeres con seguro médico no contributivo y contributivo, respectivamente. Tener un seguro médico privado se asoció con un menor uso de los anticonceptivos modernos (OR: 0,94; IC del 95%: 0,94-0,94). Las mujeres utilizaban con más frecuencia anticonceptivos como los dispositivos intrauterinos, la amenorrea de la lactancia y la ligadura de trompas.

**Conclusión:**

La prevalencia del uso de los anticonceptivos modernos en Indonesia es inferior a la meta del 75% de los Objetivos de Desarrollo Sostenible para 2030. Debido a que el seguro nacional de salud se relaciona positivamente con el uso de los anticonceptivos modernos, se recomienda ampliar su cobertura en las islas remotas de Indonesia para aumentar el uso de estos métodos anticonceptivos en esas regiones.

## ملخص

الغرض لتقييم الرابط بين تغطية التأمين الصحي والخصائص الاجتماعية السكانية، واستخدام وسائل منع الحمل الحديثة في إندونيسيا.

الطريقة استخدمنا بيانات من تعداد تنظيم الأسرة الإندونيسي لعام 2021، والذي شمل 38408597 زوجًا. يتم تغطية وسائل منع الحمل من جانب نظام التأمين الصحي الوطني: الأعضاء غير المساهمين (للأسر الفقيرة التي لا تقدم أي مساهمة مالية)، أو المساهمين (للأسر الميسورة التي تدفع مقابل التأمين). استخدمنا تحليلات التحوف لفحص العلاقة بين كل نوع من أنواع التأمين الصحي (غير قائم على المساهمات، أو قائم على المساهمات، أو خاص، أو لا شيء)، واستخدام وسائل منع الحمل، ونوع الوسائل المستخدمة.

النتائج كان انتشار استخدام وسائل منع الحمل الحديثة في إندونيسيا %57.0 (21897319/38408597). مقارنة بعدم وجود تأمين صحي، كان الحصول على تأمين صحي مرتبطًا باحتمالية أكبر لاستخدام وسائل منع الحمل، نسبة الاحتمالات OR): 1.14) (بفاصل ثقة مقداره %95: 1.13 إلى 1.14) ونسبة الاحتمالات: 1.01 (بفاصل ثقة مقداره %95: 1.01 إلى 1.01) للنساء اللاتي لديهن تأمين صحي غير قائم على المساهمات، أو قائم على المساهمات، على الترتيب. كان الحصول على تأمين صحي خاص مرتبطًا مع استخدام أقل لوسائل منع الحمل الحديثة (نسبة الاحتمالات: 0.94؛ ‏بفاصل ثقة مقداره %95: 0.94 إلى 0.94). كانت الأجهزة المثبتة داخل الرحم، وانقطاع الطمث أثناء الرضاعة، وربط قنوات فالوب هي أكثر أشكال وسائل منع الحمل التي تستخدمها النساء شيوعًا.

الاستنتاج إن انتشار استخدام وسائل منع الحمل الحديثة في إندونيسيا أقل من %75 من القيمة المرجوة من أهداف التنمية المستدامة لعام 2030. نظرًا لأن التأمين الصحي الوطني يرتبط ارتباطًا إيجابيًا باستخدام وسائل منع الحمل الحديثة، فإنه يُوصى بتوسيع نطاق تغطيته في الجزر الإندونيسية النائية لزيادة استخدام وسائل منع الحمل هذه في تلك المناطق.

## 摘要

**目的:**

旨在评估印度尼西亚健康保险覆盖范围与社会人口特征以及现代避孕药具的使用之间的关联。

**方法:**

我们使用了 2021 年印度尼西亚计划生育普查的数据，其中包括 38,408,597 对夫妇。国家健康保险计划涵盖的避孕药具：分为非缴费型（不缴纳任何费用的贫困家庭）和/或缴费型（支付保险费用的富裕家庭）。我们使用回归分析来检验每种健康保险类型（非缴费型、缴费型、私人型或无保险）与避孕药具使用和所使用的避孕药具类型之间的相关性。

**结果:**

印度尼西亚现代避孕药具的使用率为 57.0% (21,897,319/38,408,597)。与没有健康保险的夫妇相比，拥有健康保险的夫妇使用避孕药具的可能性更大，其中拥有非缴费型和缴费型健康保险妇女的比值比 (OR) 分别为：1.14（95% 置信区间，CI：1.13 - 1.14）和 OR：1.01（95% CI：1.01 - 1.01）。拥有私人健康保险的夫妇对现代避孕药具的使用率较低（OR：0.94；95% CI：0.94 - 0.94）。宫内避孕器、哺乳期闭经和输卵管结扎是妇女最常用的避孕方法。

**结论:**

印度尼西亚现代避孕药具使用率低于 2030 年可持续发展目标中 75% 的具体指标。由于国民健康保险与现代避孕药具的使用呈正相关，因此建议将其覆盖范围扩大到印度尼西亚偏远岛屿，以增加这些地区对此类避孕方法的使用。

## Резюме

**Цель:**

Оценить связь между охватом медицинским страхованием и социально-демографическими характеристиками, а также использованием современных средств контрацепции в Индонезии.

**Методы:**

В работе использовались данные переписи населения по планированию семьи, проведенной в Индонезии в 2021 году, которая включала 38 408 597 пар. Средства контрацепции покрываются национальной системой медицинского страхования: членские взносы не предусматривают внесения регулярных платежей (для бедных семей, которые не делают никаких денежных взносов) и (или) предусматривают частичные взносы (для более обеспеченных семей, которые платят за страхование). Для изучения корреляции между каждым видом медицинского страхования (не предусматривающего внесения регулярных платежей, с частичными взносами, частного страхования или его отсутствия) и использованием средств контрацепции, а также типом применяемых средств контрацепции использовался регрессионный анализ.

**Результаты:**

Распространенность использования современных средств контрацепции в Индонезии составила 57,0% (21 897 319/38 408 597 пар). По сравнению с отсутствием медицинского страхования его наличие было связано с большей вероятностью использования средств контрацепции, отношение шансов (ОШ) 1,14 (95%-й ДИ: 1,13–1,14) и ОШ 1,01 (95%-й ДИ: 1,01–1,01) для женщин со страховкой, не предусматривающей внесения регулярных платежей, и со страхованием с частичными взносами соответственно. Наличие частного медицинского страхования было связано с меньшим использованием современных средств контрацепции (ОШ: 0,94; 95%-й ДИ: 0,94–0,94). Наиболее распространенными формами контрацепции, используемыми женщинами, были внутриматочные спирали, лактационная аменорея и перевязка труб.

**Вывод:**

Распространенность использования современных средств контрацепции в Индонезии ниже целевого показателя в 75%, предусмотренного Целями в области устойчивого развития на 2030 год. Поскольку национальное медицинское страхование положительно коррелирует с использованием современных средств контрацепции, рекомендуется расширить его охват на отдаленных островах Индонезии с целью повышения использования таких методов контрацепции в этих районах.

## Introduction

Family planning, including modern contraceptive methods, is a globally recognized strategy for reducing maternal and neonatal mortality, particularly in low- and middle-income countries.^1^ As a low- and middle-income country with 273 million inhabitants, Indonesia is seeking to end population growth for long-term economic and social well-being.^2^ Indonesia’s National Population and Family Planning Board was established in 1970, and it contributed to an increased prevalence of contraceptive use of about 60.0% between 1960 and 2002.^3^ At the same time, the country halved its fertility rate from 5.6 to 2.6 births per woman. This decline, however, has stalled. Compared with other highly populous developing countries and countries in the World Health Organization (WHO) Region for South-East Asia, Indonesia’s fertility rate has not changed greatly since 2002, and was 2.18 births per woman in 2020.^4^^,^^5^ However, substantial variation exists between the Indonesian islands. For example, the fertility rate in Java and Bali is 1.98 births per woman, whereas the rates are 2.61 and 2.71 births per woman in Nusa Tenggara and Papua, respectively (Box 1).^3^^,^^6^ The country’s contraceptive prevalence increased by only 1.5% between 2007 and 2017, and its maternal mortality rate remains high at 305 deaths per 100 000 live births over the same period.^7^

Box 1 **Total fertility rate by island, 2021, Indonesia**

Sumatra: 2.32 births per woman

Java–Bali: 1.98 births per woman

Nusa Tenggara: 2.61 births per woman

Kalimantan: 2.30 births per woman

Sulawesi: 2.35 births per woman

Maluku: 2.50 births per woman

Papua: 2.71 births per woman

Total: 2.18 births per woman

Source: Indonesia family planning census 2021.^3^^,^^6^

The stagnation of the fertility rate in Indonesia is largely because of a complicated devolution process that shifted control of family planning programmes from the national level to local governments in 2001. This change resulted in an unclear understanding of the roles and responsibilities of family planning governance.^8^ An important issue of family planning services under the devolution system was that contraception services were no longer free of charge as they had been under the centralized system. Most local governments refused to pay for contraception services for their citizens. To solve this problem, the national government included contraception services within the national health insurance system in 2016.^9^

The national health insurance system, a single-payer universal health coverage system, was implemented in Indonesia as a comprehensive national insurance scheme in 2014.^10^ People covered by the scheme are enrolled in either the non-contributory (for poorer people) or contributory schemes (for salaried, non-salaried (paid by the hour or day) and self-employed workers and their family members). The government covers payments for the non-contributory scheme through national and local government budgets. For the contributory scheme, there are three classes of coverage with different fees and the cost is met by the participant of the scheme. With contraception services included in the national health insurance system, people from poor families can obtain free family planning counselling and modern contraception. The family planning services can be accessed at public and private clinics that have signed contracts with the Social Health Insurance Administration Body. This body is authorized by the government to provide health insurance programmes for the Indonesian people. With support of this body, national family planning agencies can employ family planning field workers to provide family planning education and consultation services across the country. Without health insurance, individuals would pay about 500 000 Indonesian rupiahs (equivalent to 35.7 United States dollars (US$)) for a contraceptive implant; 6–7 million Indonesian rupiahs (US$ 428.6) for tubal ligation and vasectomy;  40 000 Indonesian rupiahs (US$ 2.8) per injectable contraceptive; and 400 000 Indonesian rupiahs (US$ 28.6) for an intrauterine device. All these modern contraceptives are included in the insurance scheme and have been available free of charge to all members since 2016.^9^

Few studies have evaluated the integration of family planning services into the national health insurance scheme in Indonesia. Most studies available were conducted before the integration, and focused on certain types of health insurance and certain regions.^11^^,^^12^ Hence, the findings cannot be widely generalized. To understand the benefits of this policy in increasing the use of modern contraceptives and reducing fertility rates in Indonesia, it is important to determine the relationship between health insurance coverage and use of modern contraceptives. Thus, the main aim of our study was to identify whether the integration of family planning services into the national health insurance scheme was associated with the use of modern contraception in Indonesia.

## Methods

### Data source

We used data from the Indonesian Family Census 2021,^3^^,^^6^ which is managed by the National Population and Family Planning Board. The census collected data from 220 038 950 individuals (81.4% of the Indonesian population of 270 203 917 in 2020), 38 408 597 couples aged 15–49 years old, and 66 206 546 households from 514 districts in Indonesia. See Table 1 for the age category according to marital status in the census data. The census asked questions on: (i) sociodemographic characteristics (age, sex, marital status, religion, education, employment status and health insurance coverage); (ii) family planning (including number of children, pregnancy status, contraception use, reasons for not using contraception, type of contraception used and access to family planning services); and (iii) family development (for example, participation in *Posyandu* (community-based health services) for children younger than 5 years, family access to the internet and marriage registration number).

**Table 1:** Age group of respondents by marital status, 2021, Indonesia

Age group, years	No. (%)				
	Not married	Married	Divorced	Widowed	Total
< 19	68 963 620 (31.3)	210 671 (0.1)	14 275 (0.0)	2 106 (0.0)	69 190 672 (31.4)
19–25	19 237 935 (8.7)	5 892 708 (2.7)	221 783 (0.1)	29 273 (0.0)	25 381 699 (11.5)
26–45	9 651 377 (4.4)	54 947 578 (25.0)	2 052 950 (0.9)	888 643 (0.4)	67 540 548 (30.7)
> 46	1 376 549 (0.6)	44 935 898 (20.4)	1 833 925 (0.8)	9 779 659 (4.4)	57 926 031 (26.3)
**Total**	**99 229 481 (45.1)**	**105 986 855 (48.2)**	**4 122 933 (1.9)**	**10 699 681 (4.9)**	**220 038 950 (100.0)**

Source: Indonesia family planning census 2021.^3^^,^^6^

Note: Inconsistencies arise in some values due to rounding.

### Variables

The dependent variable for the analyses was the use (yes or no) of a modern method of contraception, that is, intrauterine device, implant, injection, lactational amenorrhoea, tubal ligation, pills, condoms and vasectomy. Respondents were asked, “Are you currently doing something or using any method to delay or avoid getting pregnant?” If respondents answered yes, the interviewer then asked, “What method are you using?”

The independent variable was the type of health insurance coverage at the time of the census, that is, non-contributory public health insurance, contributory public health insurance or private health insurance. In the census, respondents were asked, “Do you currently have health insurance?” If the respondent answered yes, the interviewer then asked, “What type of health insurance do you currently have?”

**Fig. 1:**
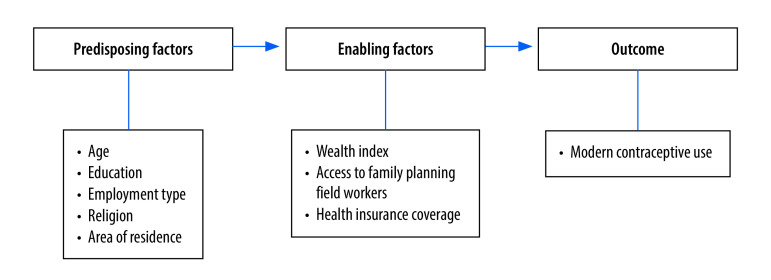
Conceptual framework of the study on health insurance and contraceptive use, Indonesia

Note: Based on Andersen and Newman.^13^

Sociodemographic, reproductive and obstetric characteristics, and previous experience with service use, are associated with contraception use. We based our conceptual framework on a previous model^13^ illustrating the predisposing and enabling factors for the use of contraceptives (Fig. 1). The predisposing factors examined in our study were age, education, employment type, religion and area of residence. The enabling factors were being in the top wealth quartile; having access to family planning field workers; and having health insurance (further information is available in the online repository).^4^

### Statistical analysis

We used descriptive statistics for the demographic findings, type of contraceptive used and type of health insurance. We used logistic regression analysis to assess the correlation between each health insurance type and contraception use. The covariates included in the logistic regression analysis were: age; employment type; education; religion; having access to family planning field workers; wealth quartile; and island of residence. We report the odds ratios (OR) and 95% confidence intervals (CI) for the logistic regression analyses. We used Stata, version 17 (StataCorp. LP, College Station, United States of America) for all analyses.

## Results

### Descriptive statistics

Among the females who used modern contraception, 43.3% (9 489 858/21 897 319) were covered by the non-contributory health insurance scheme for poor families, 21.6% (4 724 791/21 897 319) were covered by the contributory health insurance for non-poor families and 2.5% (548 058/21 897 319) had private health insurance; 32.6% (7 134 612/21 897 319) of females using modern contraceptives reported having no health insurance (online repository).^4^

Only a minority of females aged 15–25 years were covered by any health insurance, whether public or private (online repository).^4^ Of females who were not, or not yet employed (mostly housewives), 43.2% (9 151 885/21 185 747) were covered by non-contributory health insurance for poor families. A small proportion of non-employed females were covered by private insurance (2.8%; 588 417/21 185 747). About half of the females who had no formal education, and those who had completed elementary school were covered by the non-contributory health insurance for poor families: 46.9% (231 020/493 076) and 51.7% (6 414 258/12 417 580), respectively. Among Hindu (54.1%; 362 338/669 783), Muslim (41.2%; 14 338 648/34 795 672), Christian (43.9%; 861 762/1 965 213) and Catholic (46.0%; 385 323/836 973) women, the greatest proportions had non-contributory health insurance for poor families. (In Indonesia, Christian is officially separated from Catholic, and these were separate choices in the survey.) Among Confucian and Buddhist women, the greatest proportions had contributory health insurance for non-poor families – 44.5% (3507/7887) and 36.8% (44 997/122 430), respectively. Of females with non-contributory health insurance for poor families, 51.8% (8 217 677/15 981 417) had access to family planning field workers. Of females covered by the contributory health insurance for non-poor families scheme, 62.0% (5 492 643/8 851 983) lived on Java or Bali (online repository).^4^

### Distribution by district

**Fig. 2:**
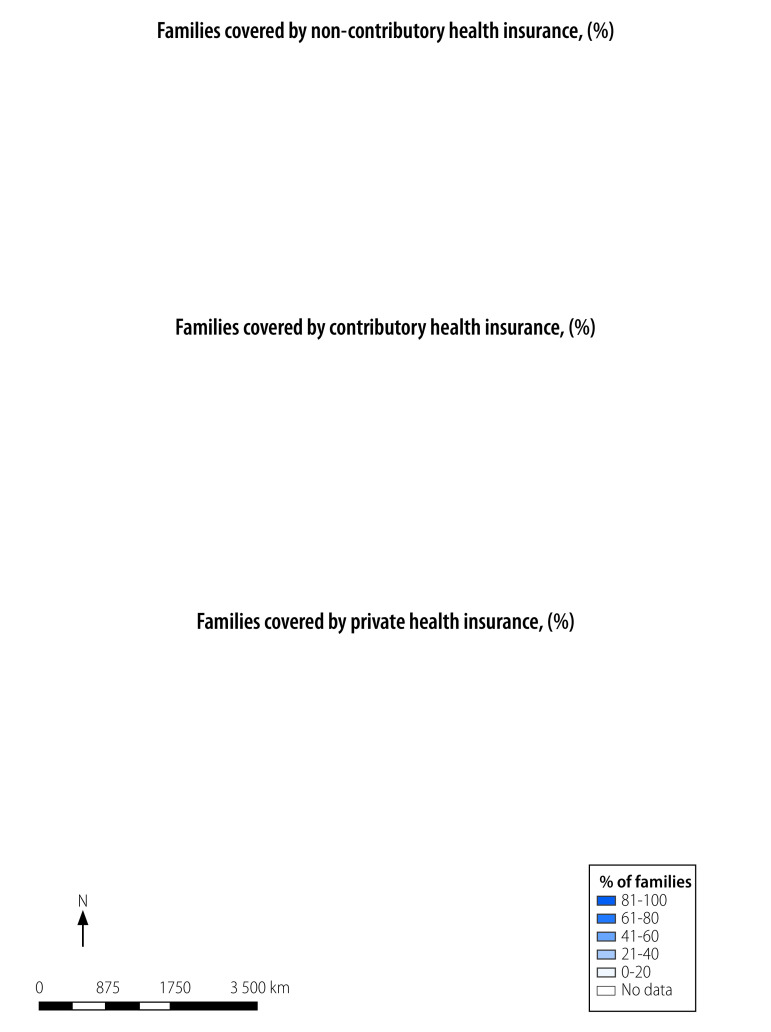
Type of health insurance coverage by district, Indonesia, 2021

**Fig. 3:**
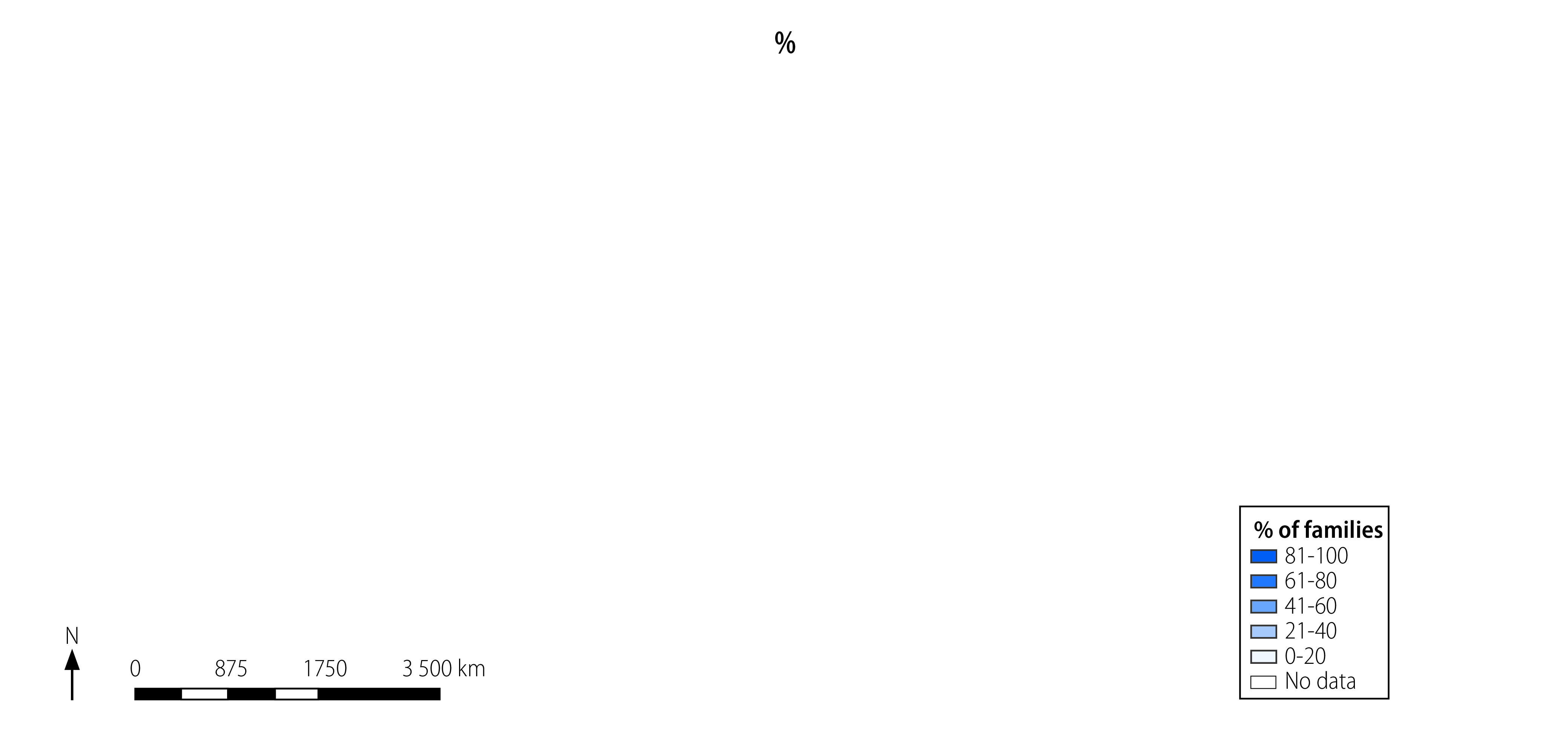
Percentage of families using modern contraception by district, Indonesia, 2021

Fig. 2 shows the spatial distribution of health insurance coverage in the 514 districts in Indonesia. Of the total sample, 41.6% (15 981 417/38 408 597) had non-contributory health insurance; 23.0% (8 851 983/38 408 597) had contributory health insurance, and 2.8% (1 070 418/38 408 597) had private health insurance coverage. The use of modern contraception varied across the districts (Fig. 3), with use in Papua lower than other main districts (see online repository).^4^

### Use of contraception

**Fig. 4:**
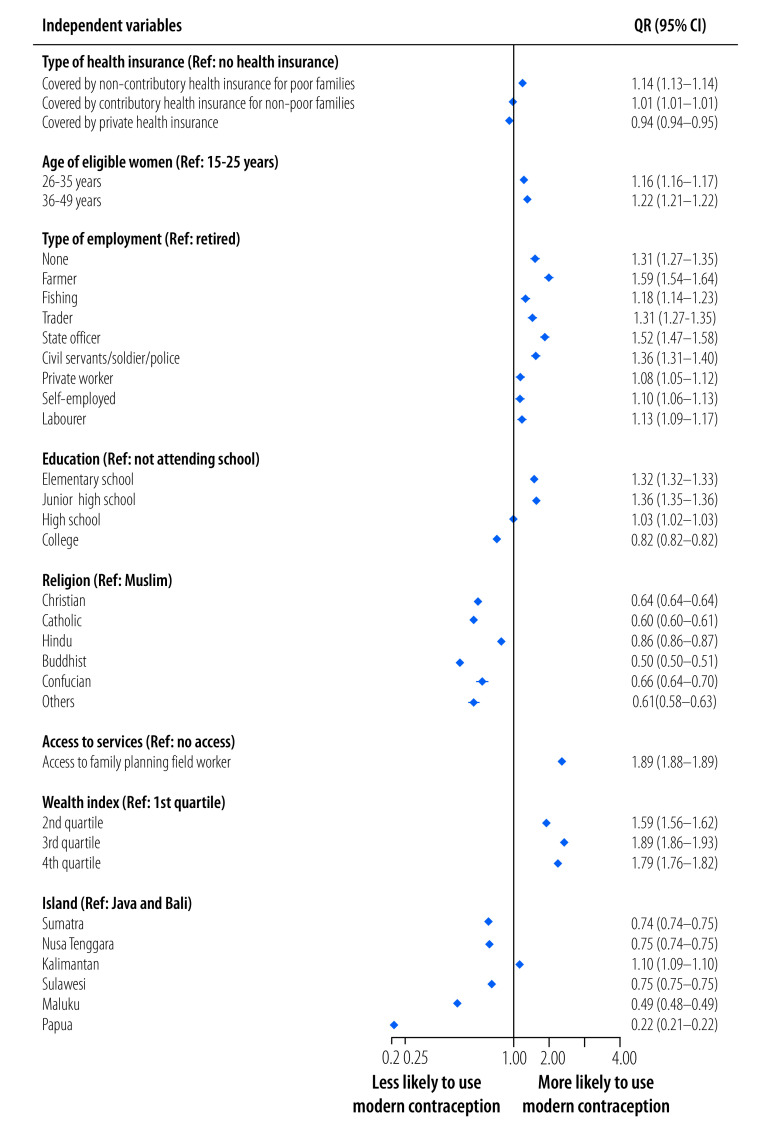
Factors associated with modern contraception use, Indonesia, 2021

CI: confidence intervals; OR: odds ratio.

Notes: The government identifies families as poor based on the assessment of neighbourhood and village heads that the income of the families does not meet their needs. In Indonesia, Christian is officially separated from Catholic, and these were separate choices in the survey.

The overall prevalence of the use of modern contraceptives in Indonesia was 57.0% (21 897 319/38 408 597). Fig. 4 shows the factors associated with the use of modern contraceptive methods for the total census population (38 408 597 eligible women). Controlling for confounding factors, women who were covered by non-contributory health insurance for poor families were 1.14 times (95% CI: 1.13–1.14) more likely to use modern contraceptives than women who did not have health insurance. Women covered by contributory health insurance for non-poor families were 1.01 times (95% CI: 1.01–1.01) more likely to use modern contraceptives than women who did not have health insurance. In contrast, women covered by private health insurance were less likely to use modern contraceptives than women who did not have health insurance (OR: 0.94; 95% CI: 0.94–0.94). Other factors associated with greater use of contraceptives were age (older than 26 years); any employment versus retirement; education up to high school versus no schooling; and second wealth quartile and above versus first quartile.

### Type of contraception used

**Fig. 5:**
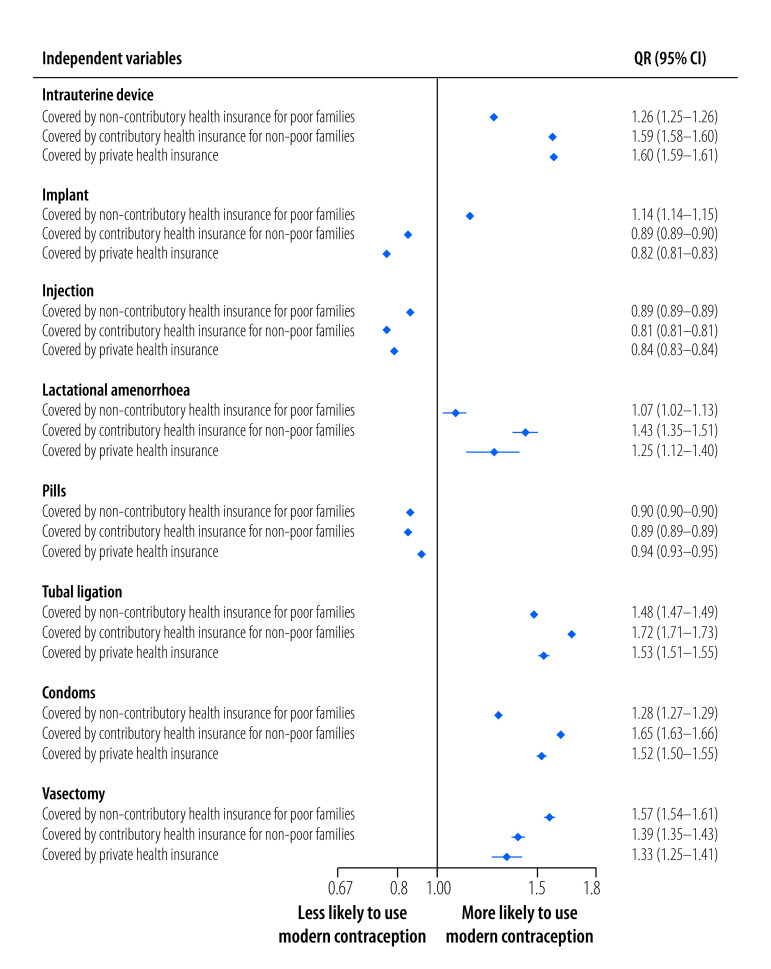
Association between type of modern contraception used and health insurance category, Indonesia, 2021

CI: confidence intervals; OR: odds ratio.

Notes: The government identifies families as poor based on the assessment of neighbourhood and village heads that the income of the families does not meet their needs.

Fig. 5 shows multivariable logistic regression analyses of the association between the use of each type of contraceptive and insurance coverage. All results were adjusted for confounding variables (results according to island of residence is available in the online repository).^4^

Compared with uninsured women, women who were covered by non-contributory health insurance for poor families were more likely to use intrauterine devices, implants, lactational amenorrhoea and tubal ligation, and less likely to use pills or injections. Among women who had contributory health insurance for non-poor families, the likelihood of using tubal ligation was greater than for other contraceptive methods (OR: 1.72; 95% CI: 1.71–1.73). Compared with uninsured women, women who were covered by contributory health insurance were also more likely to use intrauterine devices and lactational amenorrhoea but less likely to use implants, injections or pills. Women with private health insurance were also more likely to use intrauterine devices, lactational amenorrhoea and tubal ligation. In addition, these women were less likely to use implants, injections and pills.

Men with any type of health insurance were more likely to use condoms and vasectomy than men who did not have health insurance. The likelihood of using condoms was highest among men with contributory health insurance for non-poor families (OR: 1.65; 95% CI: 1.63–1.66), while the highest likelihood of using vasectomy was among men who had non-contributory health insurance for poor families (OR: 1.57; 95% CI: 1.54–1.61).

Receiving information about family planning practices from family planning field workers was significantly associated with contraception use in all islands (online repository).^4^

## Discussion

In 2016, the Indonesian government began including family planning services in the national health insurance scheme. The integration of family planning services aimed to reduce financial barriers to modern contraception services under the decentralized family planning system. The main aim of our study was to examine whether the objective of the policy was achieved. We found that both contributory and non-contributory health insurance within the national health insurance scheme was associated with the use of modern contraceptive methods in 2021.

Our findings concur with earlier studies which reported the benefits of health insurance on family planning uptake.^14^^–^^16^ Research has shown that having health insurance reduced financial barriers to and hence increased access to family planning in Rwanda and Türkiye. In Türkiye, integrated modern contraception services within national health insurance substantially reduced out-of-pocket costs, which in turn increased the use of modern contraceptives by women.^17^ A study in Rwanda confirmed the benefit of integrated family planning services in health insurance in sustaining the use of modern contraceptives.^18^

The relationships between health insurance coverage and use of modern contraceptives differed by the type of health insurance in our study. Having non-contributory health insurance for poor families was associated with greater use of contraceptives than being uninsured. In addition, the likelihood of using modern contraceptives was higher among individuals with non-contributory health insurance than among those with contributory health insurance. The results suggest that the health coverage provided by the government improves access to modern contraceptives, especially among low-income families. A study in the United States of America reported that, among low-income women, health insurance coverage was associated with higher odds of uptake of the most effective contraceptive methods or moderately effective methods.^19^ Health insurance coverage may facilitate a shift to modern contraceptives among women. We also found health insurance coverage was associated with a higher uptake of more costly methods – that is, intrauterine devices, tubal ligation and vasectomy – and lower uptake of cheaper methods such as injections and pills. By contrast, having private health insurance was associated with a lower uptake of modern contraceptive methods. This finding might be because of differences in the services that the insurance companies cover. Some private insurance companies cover the costs of contraception while others do not, potentially limiting access to services.

The prevalence of the use of modern contraceptives in Indonesia was 57.0% (21 897 319/38 408 597). This prevalence is lower than the global proportion of women whose family planning needs are satisfied by modern methods (77.0%; 0.847 billion/1.1 billion).^20^ Our findings show a prevalence of contraception use similar to the survey in the Philippines (57.2%; 2573/4497) and slightly higher than that in Myanmar (55.7%; 3925/7047).^21^ However, the national figure masks substantial variations in prevalence among provinces and districts in Indonesia, as also indicated in our study. Of the women who were using a modern contraceptive method, 59.9% (13 119 320/21 897 319) were using injectable contraceptive methods. As suggested in previous studies, women choose this method because of its convenience (it is not taken daily), comfortable administration and comparatively widespread availability. This result is in line with findings from Ethiopia.^22^ Child spacing is one of the main reasons why women use modern contraceptives, and injection is considered a convenient family planning method to achieve this goal.

Our finding that receiving information about family planning from family planning field workers had a significant influence on the use of contraceptives in all islands corroborates a previous study in Indonesia.^23^ This earlier study showed that, among young married women, discussing family planning with health workers was associated with greater use of modern contraceptive methods. A study in Nigeria also found that discussing contraceptive methods with a health worker was associated with 1.21 higher odds of using modern contraceptive methods.^24^ Health workers are influential in encouraging the use of contraceptives, as the family planning messages they deliver can highlight women’s well-being and the benefits of delaying and spacing pregnancies.^25^ Improving the quality of the communication between health workers and health-care users is important given its association with higher uptake levels and continued use of modern contraceptive methods.^26^ The Indonesian government could improve the quality of communication by increasing the salaries of family planning workers, which would enable the recruitment of better qualified personnel to encourage participation in family planning.

Our study has some limitations. First, the cross-sectional nature of the data limits our ability to draw causal inferences. Therefore, we can report only associations. Second, all assessments were based on respondents’ self-reports, which may have resulted in substantial underestimates or overestimates of contraceptives they used, which could undermine the accuracy of our estimates of the prevalence of the uptake of modern contraceptives in Indonesia. Finally, only married women (couples) were asked about the use of modern contraceptives in the 2021 Indonesian family planning census. Future studies should include unmarried or divorced women to enable the findings to be generalized to all women in Indonesia.

In conclusion, incorporating family planning services in a universal health insurance package could be an important strategy to overcome the stagnant fertility rate in Indonesia under the decentralized health system. A considerable proportion of the women in the census did not have health insurance. Thus, the government should create initiatives to encourage women and families to join the health insurance scheme. For example, the government could provide a health insurance premium that is affordable for the general public; alternatively, premiums could be calculated based on income brackets. We identified the preferred type of modern contraceptive for each type of health insurance across the islands. This information can be used by policy-makers to better tailor their programmes based on the different needs of the populations in each island.

## References

[1] World fertility and family planning. 2020. New York: United Nations Department of Economic and Social Affairs, Population Division; 2020. Available from: https://www.un.org/en/development/desa/population/publications/pdf/family/World_Fertility_and_Family_Planning_2020_Highlights.pdf [cited 2022 May 1].

[2] Indonesia population projection 2020–2045. Jakarta: Indonesia Central Bureau of Statistics; 2021. Available from: https://www.bps.go.id/publication/2018/10/19/78d24d9020026ad95c6b5965/proyeksi-penduduk-indonesia-2015-2045-hasil-supas-2015.html [cited 2022 Sep 2].

[3] [Population performance and accountability survey program for family planning and family development]. Jakarta: National Population and Family Planning Board; 2021. Indonesian.

[4] Sujarwoto S, Maharani A, Ekoriano M. Association between health insurance coverage and contraceptive use: findings from the Indonesian Family Planning Census 2021 – supplementary files. London: figshare; 2023. 10.6084/M9.FIGSHARE.22688080 PMC10388140 37529022

[5] Fertility rate, total (births per woman) – Indonesia [internet]. Washington, DC: World Bank; 2022. Available from: https://data.worldbank.org/indicator/SP.DYN.TFRT.IN?locations=ID [cited 2022 May 23].

[6] Sujarwoto S, Maharani A, Ekoriano M. Indonesia contraceptive use 2021 dataset for article title “Association between health insurance coverage and contraceptive use: findings from the Indonesian Family Planning Census 2021. London: figshare; 2023. 10.6084/M9.FIGSHARE.22650244 PMC10388140 37529022

[7] Utomo B, Sucahya PK, Romadlona NA, Robertson AS, Aryanty RI, Magnani RJ. The impact of family planning on maternal mortality in Indonesia: what future contribution can be expected? Popul Health Metr. 2021 Jan 11;19(1):2. 10.1016/S0140-6736(14)60244-0 33430907 PMC7802230

[8] Seiff A. Indonesia to revive national family planning programme. Lancet. 2014 Feb 22;383(9918):683. 10.1016/S0140-6736(14)60244-0 24567972

[9] [Regulation of Ministry of Health number 52 of 2016 regarding standards tariff for health insurance programme]. Jakarta: Indonesia Ministry of Health; 2016. Available from: https://peraturan.bpk.go.id/Home/Details/139647/permenkes-no-6-tahun-2018 [cited 2022 May 1]. Indonesian.

[10] Mboi N, Surbakti IM, Trihandini I, Elyazar I, Houston Smith K, Bahjuri Ali P, et al. On the road to universal health care in Indonesia, 1990–2016: a systematic analysis for the Global Burden of Disease Study 2016. Lancet. 2018. Aug 18;392(10147):581–91. 10.1016/S0140-6736(18)30595-6 29961639 PMC6099123

[11] Sulistiawan D, Lazuardi L, Biljers Fanda R, Asrullah M, Matahari R, Arifa RF. Who experiences out-of-pocket expenditures for modern contraceptive use in Indonesian universal health coverage system? Med-Leg Update. 2021 Jul-Sep;21(3):193–200. 10.37506/mlu.v21i3.2984

[12] Nilam Sari A, Indra Susanti A, Indraswari N, Ekawati R, Suhenda D, Nuraini. An analysis of sociodemographic, knowledge, source of information, and health insurance ownership on the behaviour of women of childbearing age in contraception use in West Java. Malays J Public Health Med. 2021 Dec 28;21(3):183–91. 10.37268/mjphm/vol.21/no.3/art.964

[13] Andersen R, Newman JF. Societal and individual determinants of medical care utilization in the United States. Milbank Mem Fund Q Health Soc. 1973 Winter;51(1):95–124. 10.1111/j.1468-0009.2005.00428.x 4198894

[14] Reichhardt DC. Leveraging antenatal care with structured contraceptive counseling to cultivate knowledge and acceptability of postpartum intrauterine methods. Phoenix: Grand Canyon University; 2020. Available from: https://www.proquest.com/docview/2435170806?pq-origsite=gscholar&fromopenview=true [cited 2022 May 1].

[15] Fagan T, Dutta A, Rosen J, Olivetti A, Klein K. Family planning in the context of Latin America’s universal health coverage agenda. Glob Health Sci Pract. 2017. Sep 28:5(3):382–98. Available from: 10.9745/GHSP-D-17-00057 28765156 PMC5620336

[16] Chen W, Zhang Q, Renzaho AMN, Zhou F, Zhang H, Ling L. Social health insurance coverage and financial protection among rural-to-urban internal migrants in China: evidence from a nationally representative cross-sectional study. BMJ Global Health. 2017. Oct 16;2(4):e000477. 10.1136/bmjgh-2017-000477 29082027 PMC5652549

[17] Celik Y, Hotchkiss DR. The socio-economic determinants of maternal health care utilization in Turkey. Soc Sci Med. 2000. Jun;50(12):1797–806. 10.1016/S0277-9536(99)00418-9 10798333

[18] Bucagu M, Kagubare JM, Basinga P, Ngabo F, Timmons BK, Lee AC. Impact of health systems strengthening on coverage of maternal health services in Rwanda, 2000–2010: a systematic review. Reprod Health Matters. 2012 Jun;20(39):50–61. 10.1016/S0968-8080(12)39611-0 22789082

[19] Kavanaugh ML, Douglas-Hall A, Finn SM. Health insurance coverage and contraceptive use at the state level: findings from the 2017 behavioral risk factor surveillance system. Contracept X. 2019 Nov 15;2:100014. 10.1016/j.conx.2019.100014 32550529 PMC7286150

[20] World family planning 2022. Meeting the changing needs for family planning: Contraceptive use by age and method. New York: United Nations Department of Economic and Social Affairs, Population Division; 2022. Available from: https://desapublications.un.org/publications/world-family-planning-2022-meeting-changing-needs-family-planning-contraceptive-use [cited 2022 Dec 1].

[21] Das P, Samad N, Al Banna H, Sodunke TE, Hagan JE Jr, Ahinkorah BO, et al. Association between media exposure and family planning in Myanmar and Philippines: evidence from nationally representative survey data. Contracep Reprod Med. 2021. April 1;6(1):11. 10.1186/s40834-021-00154-9 33789777 PMC8015027

[22] Mohammed A, Woldeyohannes D, Feleke A, Megabiaw B. Determinants of modern contraceptive utilization among married women of reproductive age group in north Shoa Zone, Amhara region, Ethiopia. Reprod Health. 2014. Feb 3;11(1):13. 10.1186/1742-4755-11-13 24490810 PMC3918182

[23] Kistiana S, Gayatri M, Sari DP. Determinants of modern contraceptive use among young married women (age 15–24) in Indonesia. Glob J Health Sci. 2020;12(13):37–48. 10.5539/gjhs.v12n13p37

[24] Ankomah A, Anyanti J, Oladosu M. Myths, misinformation, and communication about family planning and contraceptive use in Nigeria. Open Access J Contracept. 2011 Jun 21;2:95–105. 10.2147/OAJC.S20921

[25] Daniel EE, Masilamani R, Rahman M. The effect of community-based reproductive health communication interventions on contraceptive use among young married couples in Bihar, India. Int Fam Plan Perspect. 2008 Dec;34(4):189–97. 10.1363/3418908 19201679

[26] Murphy EM, Steele C. Client-provider interactions in family planning services: guidance from research and program experiences. Washington, DC: USAID, Office of Population/Research Division; 2000. Available from: https://pdf.usaid.gov/pdf_docs/Pnadw586.pdf [cited 2022 May 23].

